# A Review of Protocol Implementations and Energy Efficient Cross-Layer Design for Wireless Body Area Networks

**DOI:** 10.3390/s121114730

**Published:** 2012-11-02

**Authors:** Laurie Hughes, Xinheng Wang, Tao Chen

**Affiliations:** 1 College of Engineering, Swansea University, Swansea SA2 8PP, UK; E-Mail: 515702@swansea.ac.uk; 2 School of Computing, University of the West Scotland, Paisley PA1 2BE, Scotland, UK; 3 Chigoo Interactive Technologies Ltd, Wuxi 214028, China; E-Mail: chent@chigoo.net

**Keywords:** WBAN, energy efficient, cross layer design, QoS, protocol stack, life signs monitoring

## Abstract

The issues inherent in caring for an ever-increasing aged population has been the subject of endless debate and continues to be a hot topic for political discussion. The use of hospital-based facilities for the monitoring of chronic physiological conditions is expensive and ties up key healthcare professionals. The introduction of wireless sensor devices as part of a Wireless Body Area Network (WBAN) integrated within an overall eHealth solution could bring a step change in the remote management of patient healthcare. Sensor devices small enough to be placed either inside or on the human body can form a vital part of an overall health monitoring network. An effectively designed energy efficient WBAN should have a minimal impact on the mobility and lifestyle of the patient. WBAN technology can be deployed within a hospital, care home environment or in the patient's own home. This study is a review of the existing research in the area of WBAN technology and in particular protocol adaptation and energy efficient cross-layer design. The research reviews the work carried out across various layers of the protocol stack and highlights how the latest research proposes to resolve the various challenges inherent in remote continual healthcare monitoring.

## 1. Introduction

The increasing life expectancy in most developed nations and the inherent costs of caring for an aging population have driven the advancement of technology-based sensor devices that can remotely monitor key vital life signs. Traditional methods of monitoring the health of human patients have meant wired connectivity to static measuring equipment, usually hospital-based. This requires the patient to be static for fixed periods of time and involves the close supervision by health practitioners. Patients recovering from critical illnesses are kept in hospital for long periods of time tying up beds and health practitioners' time.

The development of wireless sensors that can be placed either inside or on the human body, coupled with the remote healthcare management of patients, has the potential to revolutionize health management in the 21st century. These systems can be deployed within hospital environments, elderly care homes, or in the patients' own home or workplace. This technology could offer step-changing improvements in patient's quality of life with positive effects on mobility and ability to conduct everyday activities.

Sensor technology incorporated as part of a Wireless Body Area Network (WBAN) can monitor key physiological signals such as blood pressure (BP), electrocardiograms (ECG), blood oxidization and blood glucose levels. The data transmitted from each of the sensors can be collected locally via a purpose built “sink” or Body Control Unit (BCU) and uploaded via a gateway to remote monitoring centers. Here the data can be remotely monitored by healthcare professionals and systems developed to track key physiological signs. The MobiHealth project [1] provides a good example of this type of architecture where a patient's specific vital signals, e.g., respiration efficiency and heart rate, are monitored via a Body Area Network (BAN). The patient can press an alarm button in an emergency and can be located via the built-in Global Positioning System (GPS). Data is stored in a Mobile Base Unit (MBU) which processes sensor data and transmits it in real time to a remote server for data analysis by healthcare practitioners. MobiSense [2] is another example of a complete WBAN system where the system is geared toward monitoring patients in the ambulatory context. MobiSense can detect body postures as well as monitor key vital life signs.

The development of suitable WBAN technology is still a relatively new research area with many inherent challenges. Communication protocols must be low power and reliable, with high levels of Quality of Service (QoS) and long battery life capability. Sensor devices need to be small and unobtrusive for patients to be comfortable wearing them. These issues and others form the subject of many open research areas.

In this study we present a review of the existing research in the area of WBAN technology and in particular protocol adaptation and energy efficient cross-layer design. The study reviews the research carried out across various layers of the protocol stack and highlights how the latest research proposes to resolve the various challenges inherent in remote continual healthcare monitoring. The aim of the study is to provide a comprehensive appraisal of relevant, existing WBAN related, low power protocol research.

The remainder of the paper is organized as follows: Section 2 introduces the WBAN concept, its architecture, standards governing body, WBAN applications in patient monitoring, and its position in wireless communication systems by comparing with other technologies. Section 3 defines the basic requirements for an efficient WBAN to be used in healthcare. Section 4 describes the existing networking standards that could be used to establish a WBAN. The protocols used in each layer of WBAN are presented in Section 5 and the cross-layer approach and cross-layer approach cases are presented in Section 6. The details of the cross-layer design schemes in the existing literature are given in Section 7. Based on the review, the challenges facing in the future design of WBANs is proposed in Section 8 and finally the paper is concluded in Section 9.

## 2. Wireless Body Area Network

### 2.1. Introduction

According to the World Health Organization (WHO) 17.3 million people died in 2008 from cardiovascular disease (CVD), heart attacks and strokes [3]. 80% of deaths took place in low and middle income countries. By 2030, WHO estimates that almost 23.6 million people would die from CVD. If the major risk factors for chronic diseases were eliminated, around three quarters of heart disease, stroke and type 2 diabetes would be prevented. Diabetes is a major risk factor for CVD [4]. Both type 1 and type 2 diabetes are closely linked to CVD, which is the major cause of deaths in people with diabetes, accounting for 50% of all diabetes fatalities. These statistics help define the context in which WBAN technology could make a major contribution to the management of life threatening diseases and increase patients' quality of life. Constant monitoring via WBAN technology has the potential to highlight key indicators in the progression of CVD and ensure adequate early warnings based on sensed data that is transmitted to health practitioners. Coupled with location awareness, fall detection technology, and patient triggered alarm notification, WBAN technology has the potential to offer a complete remote health management solution.

### 2.2. WBAN Positioning

In the spectrum of short range RF technologies, WBANs share the space with a number of short range protocols. IEEE 802.15 Task Group 6 describes a WBAN as: “*low power devices operating in or around the human body (but not limited to humans) to serve a variety of applications including medical, consumer electronics /personal entertainment and other*” [5]. A WBAN operates close to 1–2 m range or within the human body and is concerned with the connectivity of nodes operating within this space. A Wireless Personal Area Network (WPAN) is termed as a network operating up to 10 m around the human body and is normally associated with non-medical based communications such as MP3 or sports/fitness based applications. A Wireless Local Area Network (WLAN) typically has a range of up to 30 m depending on protocol type and has fixed power requirements.

**Figure 1.**
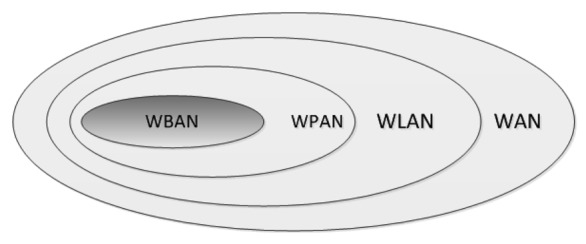
WBAN positioning.

A WBAN can be described as a specific type of Wireless Sensor Network (WSN) [6]. Figure 1 shows the context of a WBAN in relation to WPAN, WLAN and Wide Area Network (WAN). WBANs are normally associated with the human body, but not exclusively humans, where comparatively fewer nodes are deployed than traditional WSNs. With the small set of nodes, even limited data loss in WBANs could be significant, whereas data loss in a WSN is less of an issue as nodes may yield redundant information and can potentially source data from adjacent nodes. Therefore, each WBAN node needs to provide certain QoS guaranties. This may require additional measurements to ensure successful real-time data delivery, since reliability of measurements is vital in the medical domain. Both WSNs and WBANs are concerned with small footprint, low power sensing and have low data rate requirements. However a WSN is not designed for the specific set of challenges that are inherent in communications around and within the human body. This has led to a specific research focus on the medical application side of sensor networks and in particular the suitable communication protocols that are specific to WBANs.

### 2.3. Patient Monitoring & Condition Management

A WBAN can consist of a number of heterogeneous sensors and devices each having a separate role to play in the overall health management of the patient. The range of sensors that can be utilized as part of a WBAN includes ECG, blood oxidization, Electromyography (EMG), heart rate, BP, movement, and temperature [7,8].

**Figure 2.**
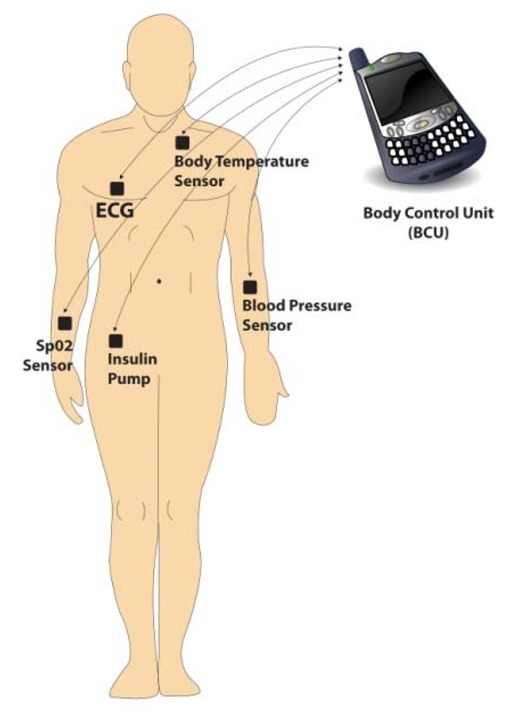
WBAN consisting of various wireless biosensors.

Sensors can be combined with actuators to form a closed loop system where sensor data is utilized to manage the levels of medicine administered to the patient. A good example of this is the potential of Continuous Blood Glucose Monitoring (CBGM) and injection in type 1 diabetes patients [9–11]. An actuator wirelessly connected to the glucose sensor via a WBAN could administer correct doses of insulin based on the sensed glucose levels of the patient at any time. This system has obvious advantages over current manual methods of self test and injection. Figure 2 shows a typical WBAN together with the range of sensors that can be linked directly to a BCU.

In addition to wearable sensors, a WBAN can include implanted sensors such as cochlear implants and various types of nanosensors that can monitor key pre- and post-operative conditions. A WBAN comprising of implantable and wearable sensors can be combined with location management, alarm notification and fall detection for a complete system that could provide the necessary levels of remote monitoring for aged and vulnerable patients who require their independence.

### 2.4. WBAN System Architectures

The role of a fully functioning WBAN is to manage the communication of a set of heterogeneous nodes placed on or inside the human body. Implanted nodes may operate at 400 MHz in the case of Medical Implantable Communication Service (MICS), whilst wearable nodes operate within the Industrial Scientific Medical (ISM) band at 2.4 GHz. Due to the low power and short range limitations of sensor devices, a “sink” or BCU in the form of a mobile phone or bespoke portable device would receive the sensor output communications from the various nodes and perform any necessary local processing. The BCU would then act as a local gateway for further communications over, e.g., Wi-Fi or General Packet Radio Service (GPRS) to a remote server for further processing and monitoring. It is assumed that a BCU would have greater energy resource than a sensor node.

**Figure 3.**
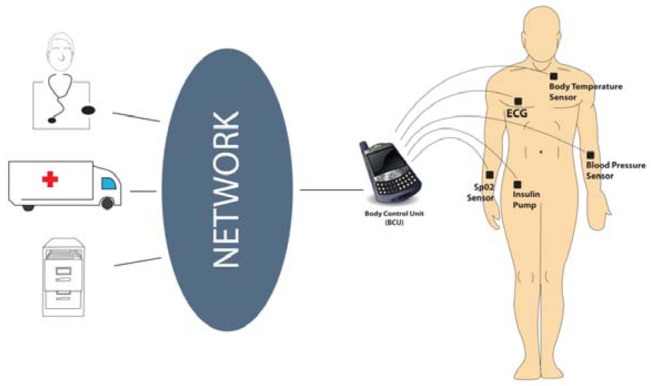
Typical WBAN architecture.

Figure 3 shows a typical BAN architecture where the personal device (mobile phone in this example) acts as the BCU to manage the data communications to the secure medical server via Wi-Fi, GPRS and the Internet. System designers must also cater for health practitioners initiating requests for key data from a specific sensor or a sensor group in priority to the normal steady state life-signs. This emergency data request could be in response to a specific health condition where a second sensor data reading is required as a priority to gain an overall picture of a potential condition. These alarm notifications would be triggered when a specific change in physiological readings is detected and highlights the closely coupled nature of individual sensors within a WBAN.

As WBAN technology becomes an integral component of healthcare management, the demands on the architecture are likely to be greater as the number and types of sensors increase along with data volumes. This means that architectures will need to cater for ever more demanding levels of energy efficiency and QoS requirements.

## 3. WBAN Requirements

### 3.1. Introduction

WBANs are concerned with the remote monitoring of key physiological signals, therefore, systems need to maintain high levels of reliability and low levels of latency. Sensor devices have relatively low data rates but energy efficiency and node battery longevity are fundamental to enable devices to maintain connectivity for long periods of time. Patients are likely to be mobile and WBANs will be processing key sensitive data that must be securely processed. Nodes are likely to be worn continuously. Patients must have confidence in the technology and be comfortable with the device on a daily basis.

### 3.2. User Experience

Nodes are required to be placed on the body at key positions to enable effective monitoring of the relevant physiological conditions. A one size fits all, easy to wear wrist-band, will not at present deliver all the functionality required to monitor key vital life signs. Therefore, patients are likely to be required to place sensor nodes on key parts of the body themselves or allow them to be fitted by health practitioners in the hospital or patients' own home or workplace. Patients' interaction with the sensors, BCU and actuators must be included as a fundamental component of the design lifecycle of WBANs. As such the WBAN must be designed to be setup by non-technical users and from a patient perspective—easy to maintain and live with. A WBAN design that makes it obvious what operations are necessary at any point in time without reference to an instruction manual will achieve greater user acceptance [12].

If WBANs are to form an integral part of the remote healthcare management of patients, they need to be robust and resilient to cope with patients' lifestyles. Patients are likely to be mobile, either within the home or work environment. This may mean basic walking, sitting and lying down, or more vigorous activities such as running and climbing stairs. WBANs operating environments are likely to vary depending on the activity and postural position of the patient. Therefore, numerous activities are likely to result in Non-Line-of-Sight (NLOS) communications between transmitter and receiver. Spectrum interference is also likely due to the proximity of other devices in the ISM band. WBANs need to be resilient enough within the standard modes of operation to ensure patients and healthcare practitioners are not faced with intermittent and unreliable monitoring results. Patients and professionals must be able to rely and trust in WBAN technology. Wearability or reliability issues that are not fixed as part of formal usability and clinical trials in a rush to market, are likely to result in patient reticence, frustration and loss of confidence in the system. The patient demographic for WBANs is likely to be biased toward the more aged sectors of the population. Fear of technology, inexperience of smart devices, mobility and dexterity issues could all be factors that may be a barrier to patient take-up. As WBAN technology moves from the research lab to the market place, designers that incorporate user experience issues are more likely to succeed.

### 3.3. Energy Efficiency

Energy efficiency within a WBAN environment is a key requirement where batteries are likely to be operational for months or years rather than days. This is especially important in the case of embedded sensors where patients may have to undergo a minor operation to change the battery. A pacemaker or glucose monitor could require a battery life of five years or more [11]. Characteristics such as low duty cycle, high levels of node sleep duration, use of efficient protocols and link adaptation/power management techniques can all contribute to extended battery life.

A node may spend large amounts of time in sleep mode but can wake up frequently to communicate key physiological data. The energy consumed during the node wakeup process can be significant and degrade battery life. Propagation and signal absorption issues can be a significant problem for WBANs requiring specific energy efficient solutions. The optimal transmission power for on-body transmission for a wireless link depends on the physical distance of the link and its instantaneous channel condition [13]. Patient mobility and posture can have a significant effect on efficient packet delivery. The inherent characteristics of RF attenuation in and around the human body can have a direct impact on channel conditions [14].

There is a potential scope for some forms of energy harvesting especially with a mobile patient, but is only likely to deliver small amounts of energy [15]. However, mechanisms that use body heat or vibration seem well suited to energy harvesting and could form part of an overall energy efficient solution [11].

### 3.4. WBAN Traffic

Sources of data traffic in a WBAN are mostly medical sensors and actuators that, depending on the physiological duty cycle, communicate key data to a BCU or sink. The nodes that communicate data around the human body mostly transmit at low data rate with fixed packets size. Most nodes transmit periodically at a regular sampling frequency [16].

Depending on key criteria, nodes within a WBAN could transmit data on a non-periodic on-demand basis. For example, a health practitioner could demand live key physiological data from a specific sensor or group of sensors in response to a monitored condition. A WBAN could be used to monitor conditions such as an irregular heartbeat where constant monitoring is required over a period of time in order to identify the relevant physiological data.

Channel access methods for WBANs need to take account of the key QoS requirements whilst being as energy efficient as possible. Time Division Multiple Access (TDMA) is a channel access method that uses a frame and slot mechanism to divide the transmission time between nodes to allow multiple nodes to transmit. Contention-based schemes such as Carrier Sense Multiple Access/Collision Avoidance (CSMA/CA) monitor the status of the channel before transmitting packets. When a channel is free the node can transmit or back-off for a fixed period before transmitting. Under high traffic levels with low bandwidth, unslotted CSMA/CA can suffer from high levels of collision resulting in increased levels of latency and energy usage. Slotted CSMA/CA, as used in 802.15.4, via the use of superframes offers an alternative approach where the communications take place within a Guaranteed Time Slot (GTS). TDMA avoids collisions but the cost of this approach is the periodic node synchronization requirement to ensure all node clocks are aligned.

Contention-based protocols such as 802.15.4 have been widely accepted in WSNs. The benefits of low energy consumption due to nodes not needing to wake up to synchronize and scalability can outweigh the drawbacks of potential increases in packet collisions over TDMA in WSNs. High levels of contention within a WBAN environment will lead to unacceptable levels of retransmission and dropped packets. This could have serious implications if key physiological data was missed. However, the high levels of traffic that could lead to collisions in a WBAN should not be critical if the volume of traffic is kept low in relation to the network transmission capacity [16].

Traffic correlation is an important characteristic in a WBAN. Each sensor node has the task of collecting and correlating sensor data. However, the probability of sensor traffic load being affected by other closely coupled nodes due to a change in a physiological condition is high [10]. For example if a patient falls ill due to fever, his body temperature will rise together with heart rate and possibly BP. These changes may be reflected in the blood oxidization levels of the patient. This close coupling of sensor nodes and their resulting behaviors due to changing physiological conditions has an impact on channel traffic. Many nodes may initiate transmission requests at the same time resulting in a burst of data and a series of concurrent medium access requests.

### 3.5. Quality of Service

QoS is accepted as a measure of the service quality that the network offers to applications and users. Network reliability and guarantees of successful transmission are vital in WBANs due to the criticality of the data [17]. WBANs share many of the QoS issues that are inherent in WSNs: *Resource constraints*: energy, bandwidth, memory, limited transmission power; *Unbalanced traffic*: Potential *n* number of nodes communicating with a sink; *Heterogeneous nodes*: variety of sensor node types; and *Packet criticality*: priority traffic based on node criticality and condition type.

QoS levels within a WBAN environment take on new levels of criticality over and above those for WSNs due to the monitoring of key physiological life signs. Reliable transmission from sensor nodes to BCU and then potentially to remote healthcare professionals over HTTP or GPRS is vital and could be life threatening if not delivered with some guarantees. QoS requirements within a WBAN include high Packet Delivery Ratio (PDR), low levels of packet transmission delay, minimal collisions and retransmissions and efficient balancing of high energy efficiency and reliable transmission. Optimized energy efficiency requires an energy management process that efficiently balances QoS with energy efficiency, adapting to heterogeneous node constraints and wireless channel dynamics. QoS can only be evaluated at the higher layers of the protocol stack while energy consumption largely appears at the lower layers. The QoS for a WBAN solution can provide the core requirements guarantees for any network design where the various protocol stack parameters can be modified and adapted to deliver efficiencies as long as the QoS levels are still fulfilled.

### 3.6. Security

WBANs must incorporate adequate levels of security and privacy to protect the sensitive nature of personal and medical related data. WBANs are vulnerable to the same forms of attacks as other wireless networks such as eavesdropping and Denial of Service (DoS). These threats can expose a WBAN to serious security vulnerabilities and affect its performance at the physical, data link, network, and transport layers [18]. Patient data must be stored locally and securely transmitted to the server side in the context of transmitting data to a remote health professional. WBAN designers need to ensure that the processes for system setup and day to day operation do not introduce security risk as these tasks will be undertaken by non technical personnel. Efficient cryptographic techniques need to be used to mitigate the risk of security breaches but must not add significant overhead to packet size or transmission times.

## 4. Existing Standards

### 4.1. Introduction

The main challenge in designing standards for WBANs is the balance of QoS demands with the low power constraints of battery powered nodes. A number of low power standards have been used in WSN/WBAN research as well as for commercial applications, all of which go some way in partly satisfying the requirements for life signs monitoring. Some low power standards designed to support low power sensing have been adapted for healthcare applications, e.g., ZigBee, while others such as 802.15.6 have been designed specifically for WBANs and cater for personal entertainment applications as well as healthcare.

### 4.2. IEEE 802.15.4 and ZigBee

**Figure 4.**
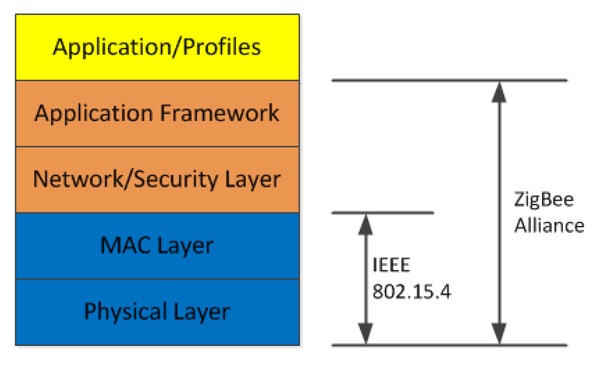
Simplified ZigBee/802.15.4 protocol stack.

ZigBee [19] is a standard designed for low power, minimal cost and extended battery life applications. The standard incorporates an application layer to define an application framework to support a number of application profiles and a network layer to implement networking and security. ZigBee has adopted 802.15.4 as its physical and MAC layers to make a complete protocol stack implementation. A simplified ZigBee protocol stack is shown in Figure 4. The application profiles include a number of standards including Building Automation, Smart Energy, Home Automation and Healthcare. The Healthcare profile enables specific health monitoring applications to use the ZigBee protocol stack. 802.15.4 operates in three frequency bands: 868 MHz (Europe), 915 MHz (ISM USA) and 2.4 GHz (ISM worldwide). The 2.4 GHz ISM band has the maximum data rate of 250 kb/s operating across 16 channels. Modulation at 2.4 GHz uses Orthogonal Quadrature Phase Shift Keying (O-QPSK) where the digital data resides in the phase of the signal. 802.15.4 uses a spread spectrum method—Direct Sequence Spread Spectrum (DSSS) to improve receiver sensitivity and reduce effects of multipath. 802.15.4 uses the concept of a Fully Function Device (FFD) and a Reduced Function Device (RFD) as the node roles.

A FFD is normally connected to a wired power supply and is capable of performing all the tasks in the 802.15.4 standard and can communicate with any device in the network. A RFD is battery powered node that is deliberately designed with limited functionality to extend battery life. Channel access in 802.15.4 can operate in beacon enabled mode via the use of superframes (slotted CSMA/CA) or in non-beacon mode (unslotted CSMA/CA).

Research has shown [20–22], that although 802.15.4 has been proven as a suitable standard for multihop WSNs providing QoS and adequate data rates, it is not the best solution for low power communications in WBANs. The technology was not designed to support WBANs and any WBAN implementation using 802.15.4 will ultimately have to adapt to the compromises necessary to deliver an efficient implementation.

### 4.3. IEEE 802.15.1 Bluetooth Low Energy

Bluetooth Low Energy (BLE) [23] has been designed as a low power WSN standard to operate in the same 2.4 GHz range as classic Bluetooth but uses different channels and a lower data rate of 200 kb/s rather than the 1–3 Mbps achieved via classic Bluetooth. BLE does not support mesh networking and only supports one packet type. BLE uses DSSS as the spread spectrum method and supports three separate application profiles: Sporting, Consumer and Healthcare. BLE is not backward compatible with classic Bluetooth, but Bluetooth 4.0 dual mode capability supports a direct interface between the standards where the protocols share one physical radio and antenna. Due to the plethora of Bluetooth-enabled devices in the marketplace, BLE is likely to be adopted widely by device manufacturers as it is a “least effort” forward path to deliver low power functionality. Although the likely wide adoption of BLE as a low power WSN standard cannot be ignored, the standard does not support QoS and is not specifically designed for WBAN applications.

### 4.4. IEEE 802.15.6

The 802.15.6 standard [5] aims to support WBAN requirements via low complexity, low cost, low power, and reliable transmission with data rates up to 10 Mbps. The standard defines a MAC layer and several physical layers. It uses the standard ISM bands as well as approved frequency bands specific to national regularity authorities. The 802.15.6 task group seeks to establish a low power communication standard while minimizing the Specific Absorption Rate (SAR) across the range of human body types (fat, thin, male, female, *etc.*). The MAC layer can be defined as beacon or non-beacon enabled modes. In non-beacon mode, the frame can be allocated as “with” or “without” superframe boundaries. In beacon mode the channel is divided into superframe structures where each superframe is bounded by a beacon. Nodes contend for channel access using CSMA/CA or slotted Aloha. Extended Access Phase (EAP) is used for high priority emergency traffic and Random Access Phase (RAP) is used for regular non-emergency traffic.

The supported frequency bands are grouped into Medical Implanted Communications (MIC), Wireless Medical Telemetry Services (WMTS) and ISM. MIC and WMTS bandwidths are not suitable for high data rate applications. The ISM band shares the spectrum with other wireless standards, e.g., 802.11 and 802.15.4 as well as household and industrial devices therefore, can be subject to interference.

**Figure 5.**
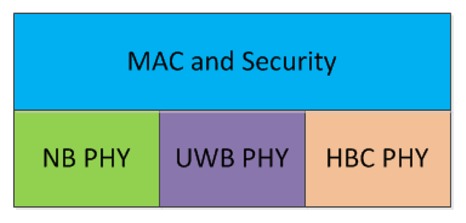
IEEE 802.15.6 high level architecture.

802.15.6 defines three physical layers, as shown in Figure 5: Narrowband (NB), Ultra Wideband (UWB) and Human Body Communication (HBC). The NB physical layer is responsible for Clear Channel Assessment (CCA), radio transceiver activation/deactivation and data transmission and reception. The Physical Protocol Data Unit (PPDU) frame of NB physical layer contains a Physical Layer Convergence Procedure (PLCP) preamble a PLCP header and a Physical Service Data Unit (PSDU). The NB physical layer does not support high data rate applications.

The UWB physical layer operates in both low and high frequency bands. The low band consists of three channels (1–3). Channel 2 (mandatory channel) has a central frequency of 3,993.6 MHz. The high band has eight channels (4–11) where channel 7 (mandatory) has a central frequency of 7,987.2 MHz. The UWB physical layer transceivers cater for low complexity and power levels similar to the MIC band [24]. The HBC physical layer operates in the 16 MHz and 27 MHz bands. Both bands are valid for the USA, Japan and Korea, whereas the 27 MHz band is approved for use in Europe. The HBC specification covers the entire protocol for WBAN including packet structure, modulation and preamble/SFD.

Although 802.15.6 has been specifically designed for WBAN applications, as the standard caters for medical and non-medical applications, it cannot deliver optimal low power performance for life signs monitoring applications. Timmons and Scanlon [25] developed MedMAC, a MAC protocol, for ultra low power WSN applications. In experiments where MedMAC was compared with 802.15.6, it was found that MedMAC displayed a marked improvement in energy consumption with an energy saving of between 25.6%–33.2% for packet rates ranging from 1–10 kb/s.

## 5. WBAN and the Communications Protocol Stack

### 5.1. Introduction

This section outlines a number of protocols operating at different layers. Many of the schemes have been outlined in the literature as WSN-specific, but are included here as either they form an important part in the understanding of sensor networks generally, or have formed the basis of WBAN specific protocols.

The unique attributes of a WBAN and its close proximity to the human body (in the context of low energy efficient communications) mean there are specific implications at each layer of the protocol stack [16]. Protocols working at each of the layers need to contribute to low packet transmission delay, minimum jitter, extremely low levels of congestion, low energy usage, whilst fulfilling specific QoS demands.

### 5.2. Physical Layer

Communications in and around the human body highlight a unique set of challenges that are not present in other types of networks. At the physical layer, protocols need to take account of how the body reacts to RF communications and the complications therein. The characteristics of RF signals at the physical layer can be separated into signal propagation effects in and along the human body and how body movement has an impact on the transmission of RF signals.

#### 5.2.1. Characteristics of RF Communications in and along the Human Body

In his work on Personal Area Networks (PAN), in particular body impedance, Zimmerman [26] concluded that the human body behaves as an almost perfect conductor but the low internal body resistance is insulated from the PAN nodes by skin, hair and clothing which collectively exhibit a large impedance level. Due to the human body composition of varying amounts of water, skin and tissue, the propagation of electromagnetic signals along or in the human body is inherently variable and subject to fading and multi-path propagation. Various organs and tissues exhibit different characteristics when RF signals are transmitted through them resulting in unpredictable outcomes in the context of absorption, propagation and required power levels.

On-body signal propagation consists mainly of a creeping wave diffracted from human tissue and trapped along the body surface [27]. RF signal behavior can vary between outdoor and indoor environments where reflection and propagation factors differ, for example a signal transmitted at the chest and reflected by a wall a few meters away will arrive at the spine based receiver with about the same power as a creeping wave. Optimal transmission power required for an on-body wireless link between two sensors depends on the distance of the link and its instantaneous channel condition. The distance can vary with mobility driven by human postures and the channel can vary due to unpredictable RF attenuation [13]. Generally, the propagation wave rather than passing through the human body tends to diffract around it. When transmit and receive antennas are placed on different sides of the body, path loss can be significant. In investigating the mobile path loss for on-body UWB links within hospital environments, Catherwood and Scanlon [28] concluded that Received Signal Strength (RSS) depended on whether transmit and receive antennas are in relative Line of Sight (LOS) or NLOS conditions and that NLOS had a significant impact on RSS. Additionally, the research showed that RSS significantly varied depending on posture position of a patient either sitting or standing.

Nodes placed on or in the human body communicate via antennas. One of the main issues in the design of antennas for use in the WBAN environment is catering for how the human body interacts with electrical and magnetic fields. Antenna design is affected by patients posture, skin type and body mass. Antenna designs should consider the intrinsic on-body environment, restrictions on size, shape and material. For antennas that are placed inside a human body, only non-corrosive and bio-compatible material, such as platinum or titanium can be used for implants. However, these materials yield a poorer performance compared to a copper antenna. The shape and size of an implant antenna depends on its location inside the body, which further limits the freedom of the designer. Heating effects on fat, muscle and skin tissue need to be considered as part of antenna design [16].

In experiments researching the effects of body shape and gender on body channels, Di Franco *et al.* [29] observed that females suffer less path loss than males, but concluded that this could be due to distance variations between wrist and torso in women. Additionally his research showed that males suffer more fade than females and the weak correlation observed between distance and path loss indicates that the shadow effect due to different body shapes has much more influence on path loss. Zasowski *et al.* [30] observed that the channels around the human torso are strongly dependent on the transmission medium. It was shown that echoes off both arms can change the attenuation from the left to right side of the body by up to 20 dB, depending on the position of the arms. The torso, being the largest part of the human body, can have a significant impact on the attenuation of RF signals. Reusens *et al.* [31] characterized the physical layer including an estimation of the delay spread, path loss and mean excess delay between two nodes on the body for NB communications. The research reviews the propagation channel between two half wavelength dipoles at 2.45 GHz, placed near a human body. A total of 583 measurements were performed in a multipath environment on real humans considering different parts of the human body: arms, leg, torso and back. Results showed that path loss increases with antenna separation and the loss models of the arm and leg are similar. The researchers found that the path loss along the torso follows the same course as the path loss along the arm but the losses along the torso are the highest of all body parts. It was concluded that this was probably due to the higher absorption in the trunk due to the larger volume and because the surface of the trunk is less flat than the surfaces of the other investigated body parts. The research in [31] did not take body movement into account, which therefore, limits the significance of the research for real life scenarios.

#### 5.2.2. Body Movement and RF Communications

The movement of the body can have a large impact on the successful transmission of RF based signals with significant loss where limbs cross the path of transmitter and receiver. Ylisaukko-oja *et al.* [32] observed a stark variance in path loss when comparing an indoor static scenario with an outdoor test where a user was conducting a number of activities (walking, running, cycling, *etc.*). Results showed greater losses in the scenarios involving movement. Quwaider *et al.* [14] experimented with the effects that posture can have on RF communications. The researchers used a closed loop transmission and power assignment framework for on-body wireless links. Node adaptive power management used Received Signal Strength Indicator (RSSI) as an additional parameter when measured between sensor and sink. The experiments included subjects being asked to follow a sequence of movements corresponding to four different sitting positions: arms to the side, arms crossed, arms raised, and hand holding one knee. The hand positions represented natural sitting postures with varying degrees of RF attenuation due to signal blockage and sensor node movements.

Link quality was characterized by observing the RSSI values at the sink node for a given static transmission power from the arm mounted sensor node. The variation of RSSI represents the changes in link quality as a result of body movements and channel condition changes due to unpredictable RF attenuation. This can be caused by subjects clothing, physical stature, and antenna orientation. In the analysis of Packet Delivery Ratio (PDR) and Energy Per Packet (EPP) levels, results showed that varying postural position could have a dramatic effect on RSSI levels. Adapting the power assignment based on received signal strength levels can provide better packet delivery and energy efficiencies in comparison to static energy schemes.

### 5.3. MAC Layer

Extensive research has been carried out on the adaptation of the MAC layer for WSNs to improve channel management and extend battery life. Although many of the schemes designed for WSNs focus on issues relating to distributed low power ad hoc networks, some have the potential to deliver improvements in WBAN applications. MAC layer research specific to WBANs is a relatively recent research area and is still an active topic for protocol designers. The transmission of RF signals is a major overhead for the energy efficiency of a WBAN. The MAC layer controls the channel access, packet encoding, addressing and as such must achieve maximum energy efficiency and data throughput by the efficient management of these functions. Studies reveal [33] that energy wastage in existing MAC protocols occurs from four major sources: *excessive collisions*, *control packet overheads*, *idle listening*, and *overhearing*. All contribute to network delay and energy inefficiency. WBAN applications require MAC protocols that are energy efficient and able to deliver battery lifetimes of several months or in the case of implanted nodes-years. MAC duty cycling needs to deliver energy efficient channel access to reduce packet collisions and reduce the impacts of idle listening and overhearing.

#### 5.3.1. MAC Mechanisms

Channel access mechanisms can be grouped into: *Schedule-based*: predominantly TDMA but also Code Division Multiple Access (CDMA); *Contention-based*: usually CSMA/CA; and *Hybrid Schemes*: combining contention and scheduled methods as part of an adaptive scheme [34].

In a schedule-based system such as TDMA, the channel is split into fixed or variable time slots with a fixed frame length that is assigned to nodes that wish to communicate. Collision is avoided as nodes can only transmit during their allocated time slot thereby avoiding any collision and wasted energy. Each of the nodes must be synchronized using separate control packets to ensure nodes send their packets in the allocated time slot. In some applications, schedule-based protocols can be more efficient using less energy due to the reduced radio duty cycle, but they do have the overhead of requiring frequent synchronization. This overhead could be significant in large networks where overall network synchronization could be problematic. Other issues with schedule-based schemes include: assigning slots to nodes and identifying the collision free slot [34].

In CSMA/CA there is no reservation of the channel. Each node will initially listen to the channel, check it's free or not then transmit. If the channel is busy the node will back off for a period of time before trying again. Contention-based schemes such as CSMA/CA can suffer from the hidden node problem together with node idle listening and packet collision. This could result in CSMA/CA incurring significant overhead in WSNs and WBANs. The main advantages of contention-based approaches are low complexity, ability to adapt to fluctuations in traffic load, and low infrastructure implications. However contention-based protocols can suffer from overhearing, idle listening and collisions, all of which will have an energy cost implication. Additionally, QoS levels cannot be guaranteed in a contention-based channel unless the number of nodes and traffic levels are stable in relation to the available bandwidth.

Comparisons between contention and schedule schemes shown in Table 1 highlight variances between the schemes depending on the performance metric. Although these sorts of comparisons are useful in an overall sense in terms of understanding the constraints of each method, the performance of each is tightly coupled to the specifics of the application environment and traffic characteristics of the network.

Hybrid schemes aim to improve the performance of the MAC layer by combining schedule- and contention- based schemes, e.g., TDMA with CSMA/CA. This type of architecture [34] can improve performance over existing schemes by adapting to network traffic conditions as they arise. A hybrid MAC can function in contention-based scheme mode when traffic is light and convert to a reservation based scheme when traffic is heavy. Simulation results show that hybrid schemes can outperform both contention- and schedule-based MAC, as they are more able to adapt to increased traffic loads thereby using less energy in the process.

#### 5.3.2. MAC Layer Adaptation Schemes

One of the most referenced low power MAC protocols in the literature is Sensor-MAC (S-MAC) [33]. S-MAC is a synchronous protocol where the basic idea is for nodes to sleep periodically and have each node somehow aware of all other nodes sleeping patterns. S-MAC is designed to save energy by periodically switching between active and sleep states, thereby making tradeoffs between energy and latency according to traffic conditions. Energy consumption is minimized by utilizing a synchronization mechanism that maintains a common sleep schedule between neighbor nodes in low duty cycle operations. S-MAC uses Request to Send (RTS)/Clear to Send (CTS) and a virtual carrier sense technique similar to 802.11 to avoid overhearing and provide adaptive listening. The protocol presents a message passing technique that separates a long message into small fragments and transmits them in a burst to loosely coupled nodes. S-MAC uses three techniques to achieve low power duty cycling: *periodic sleep*, *virtual clustering* and *adaptive listening*. S-MAC delivers significant energy improvements over 802.11 but the duty cycle is required to be synchronized to a specific traffic load. Thus its performance suffers under varying traffic loads.

Timeout-MAC (T-MAC) [35] introduces an adaptive duty cycle that dynamically ends the active part of the cycle to reduce the energy wasted on idle listening. T-MAC improves the design of S-MAC by shortening the active period if the channel is idle. In S-MAC the nodes will remain awake through the entire active period even when not receiving or transmitting data. T-MAC improves on S-MAC by listening to the channel for only a short time after the synchronization phase. The node will return to sleep mode if no packet is received during this window. T-MAC also uses an adaptive listening technique where nodes are able to immediately pass the data avoiding the timeout introduced in S-MAC. This adaptation of the duty cycle improves the energy and throughput performance under varying traffic loads. T-MAC uses one fifth of the energy used by S-MAC. However these gains come at the cost of reduced throughput and increased latency. Lu *et al.* developed DMAC [36] and identified inefficiency in S-MAC and T-MAC concerning idle listening where nodes remain active or asleep for longer than they need to. The protocol identified efficiency (low latency and low energy) in WSNs using data gathering trees. DMAC was designed to solve the interruption problem where nodes that are out of hearing range of both sender and receiver are unaware of ongoing data transmissions and will, therefore, go to sleep until the next cycle. DMAC allows continuous packet forwarding by giving the sleep schedule of a node an offset that depends upon the depth of the tree. DMAC also adjusts the duty cycles adaptively according to the traffic load in the network. Simulation results show that DMAC achieves both energy savings and low latency in comparison to other protocols when used with data gathering trees in WSNs.

Some MAC protocols such as Berkeley-MAC (B-MAC) [37] and WiseMAC [38] use the preamble element of a packet to adapt the duty cycle to ensure nodes only wake up when absolutely necessary. B-MAC is an example of an asynchronous technique where the protocol appends a long enough preamble to the data packets to ensure the destination is active at least once while the preamble is transmitted. B-MAC is a simple CSMA-based protocol that utilizes low power listening and an extended preamble to deliver efficient power management without the need for synchronization. As in S-MAC and T-MAC, nodes have an awake and a sleep period, but in B-MAC if a node wishes to transmit it precedes the data packet with an extended preamble that is slightly longer than the sleep period of the receiver. During the awake period, the channel is sensed and if a preamble is detected the node continues in awake mode to receive the data. B-MAC suffers from the overhearing problem and also suffers from an introduced overhead as all nodes in the surrounding area must listen to the long preambles. WiseMAC is a contention-based MAC protocol that performs best in channels with low or variable traffic. WiseMAC's wake up scheme consists of short periodic duty cycles that enable the nodes to sense the carrier for a preamble signal. All nodes in the network sample the medium with a common basic cycle duration *T*, but their wake up patterns are independent and unsynchronized. WiseMAC ensures all nodes learn the sampling schedule of the network and store this within a table at each node. As each node is aware of the overall schedule, a node initiates transmission at the right time with a wakeup preamble of minimized duration *T*p.

The use of the preamble sampling technique and short wake-up preamble in WiseMAC can mitigate the overhearing problem in periods of high traffic. WiseMAC showed that for the same delay, preamble sampling lowered power consumption by 57% when compared to 802.15.4. WiseMAC solves many of the issues associated with low power channel access but does not provide a mechanism by which nodes can adapt to changing traffic patterns. Buettner *et al.* [39] highlighted a number of disadvantages of using a long preamble in low power listening:

- Suboptimal energy consumption at both sender and receiving nodes.
- Overhearing issues, causing excess energy consumption at non-target nodes.
- Excess latency at each hop as nodes need to wait for the end of the preamble to either check if the packet is for them or to receive the data. The problem is magnified over a multi-hop path.

X-MAC is an improvement on the concepts of B-MAC where the protocol reduces the length of the preamble by the addition of key data that negates the node listening to the full preamble as in B-MAC. X-MAC avoids overhearing by inserting the destination address in the preamble. Nodes listening to a part of the preamble detect that the data packet is not intended for them (early acknowledgement) and go back to sleep. The shortened preamble approach used in X-MAC reduces energy usage at both transmitter and receiver nodes, reduces per hop latency and offers advantages such as flexible adaptation to both bursty and periodic data sources.

Network coding allows the intermediate nodes in a WSN to not only forward data packets but to perform limited processing on incoming packets before being transmitted to the destination node. The concept has been shown to improve the performance within WSNs by increasing throughput via the transmission of more information with fewer packet transmissions [40,41]. Antonopoulos *et al.* [40] propose a Network-Coding based Cooperative Automatic Repeat reQuest (ARQ) MAC protocol for WSN (NCCARQ-WSN) that uses relay nodes to coordinate retransmissions. The protocol is CSMA based and is therefore, compatible with 802.15.4. The protocol uses less control packets than comparable ARQ based protocols whilst delivering increased energy efficiency without compromising QoS. NC-PAN [42] is a TDMA based hybrid cooperative network coded protocol that takes advantage of ARQ techniques. The protocol exploits relaying phases to serve node traffic via network coded transmissions. Performance gains of up to 35% have been identified in terms of throughput and latency when compared to other cooperative schemes such as B-PAN and C-PAN. A summary of the key characteristics of the MAC protocols mentioned above is shown in Table 2.

#### 5.3.3. WBAN Specific MAC Layer Schemes

Existing research on energy efficient MAC protocols specific to WSNs, e.g., S-MAC, T-MAC, and DMAC, *etc.*, propose solutions to issues such as overhearing, idle listening, collisions, and increasing levels of energy efficiency. S-MAC reduces energy usage at the cost of increased latency. T-MAC improves upon S-MAC by introducing an adaptive duty cycle but introduces additional delay due to an aggressive sleep schedule. DMAC also has an adaptive duty cycle and attempts to solve the node interruption problem. Although all these protocols offer improvements to the standard MAC, they suffer from synchronization overhead, periodic exchange of sleeping schedules and are designed specifically for multi-hop WSNs. Additionally, traditional MAC protocols designed for WSNs focus on improving fairness, latency, bandwidth utilization and throughput. These areas are secondary for MAC protocols specific to WBANs where greater energy efficiency and QoS are keys.

Zhen *et al.* [43] researched the networking issues of implanted communications using 802.15.4. The research proposed that CSMA/CA cannot be adopted in WBANs due to the rapid attenuation of electromagnetic waves through tissues and unreliability of Clear Channel Assessment (CCA) in the context of implanted nodes. The CCA range was found to be of the order of 0.5 m and required the use of a body forwarder to transmit longer distances. The addition of a body forwarder led the researchers to conclude that communications between nodes could only be actioned via a minimum of two hops coupled with a low complexity routing algorithm.

However, the research focused solely on implanted nodes and didn't compare results with on-body sensors. Additionally, no account was made of varying body types and the effect this can have on multi-path propagation in and around the human body.

MedMAC [25] aimed to improve the performance of 802.15.6 for ultra low data rate WSNs/WBANs by achieving energy efficiency through a novel synchronization mechanism. Ideally, implanted devices should have ten or more years of operation life between battery changes or utilize energy harvesting technology to ensure the longevity of devices. MedMAC incorporated an Adaptive Guard Band Algorithm (AGBA) with Drift Adjustment Factor (DAF). This algorithm allowed the nodes with ultra low data rates to save power by sleeping through beacons they would normally receive to synchronize with the network. The key advantage over MAC protocols which use guard times is that the Guard Bands (GBs) are not fixed at some arbitrary or maximum level which results in unnecessary power waste. AGBA generates GBs proportional to the time elapsed from the previous synchronization point. A further refinement to the algorithm is introduced by using the DAF, which allows GBs to be based on actual drift (AD) of the respective time bases. This ensures even less waste by keeping GBs as close to the required minimum as possible. The protocol simulations showed that energy efficiencies were identified through nodes remaining synchronized while only waking when absolutely necessary. Results showed energy savings of up to 87% over 802.15.6 for the selected scenarios.

Fang *et al.* proposed BodyMAC [44], a MAC protocol that gives flexible bandwidth allocation to improve node energy efficiency by reducing packet collisions, lowering transmission times, idle listening and control packet overhead. BodyMAC is based on a downlink and uplink scheme in which the free part of the uplink subframe is completely collision free. Three bandwidth management schemes are proposed: *Burst Bandwidth*, *Periodic Bandwidth and Adjust Bandwidth*. An efficient sleep mode is introduced to reduce the idle listening duration especially for low duty cycles. Simulation results show a superior performance compared to 802.15.4. Omeni *et al.* [45] introduced an energy efficient MAC for WBANs based on single hop and central node based synchronization as a means to move network complexity away from distributed nodes and onto the central node. Once a node has joined the WBAN, collision is prevented within a cluster as all communications are initiated by the central node and addressed uniquely to a slave node. The main goal of the protocol is to reduce power consumption due to idle listening, overhearing, and collision using a CCA algorithm based on a Listen Before Transmit (LBT) and a “wakeup fallback” time to handle time slot overlaps. The disadvantage of the protocol is increased complexity at the central node although it is assumed the central node has more power and processing resources.

Body Sensor Network MAC (BSN-MAC) [46] is an 802.15.4 based ultra low power adaptive MAC designed to exploit feedback information from nodes to deliver increased energy efficiency. To avoid collisions with nearby nodes, the protocol incorporates a CCA algorithm based on LBT. The concept of a wakeup fallback time is introduced to handle time slot overlaps. Energy efficiency over 802.11 and ZigBee is realized using single hop and centrally controlled sleep/wakeup times. The control algorithm enables the BSN coordinator to adjust parameters of the 802.15.4 superframe structure to avoid idle listening and achieve both energy efficiency and low latency on energy critical nodes. As in BSN-MAC, Distributed Queuing MAC (DQ-MAC) [47] uses a superframe structure to deliver further energy efficiency as a function of network load and packet length. DQ-MAC uses BSN-MAC and 802.15.4 as a benchmark to highlight specific energy saving improvements. DQ-MAC grants immediate access for light traffic loads (behaving as a random access mechanism) and seamlessly moves to a reservation system for high traffic loads, eliminating collisions for all data transmissions.

The DQ-MAC superframe is bounded by a feedback packet (FBP) contained in the feedback frame. The FBP is used to synchronize the nodes to the BAN coordinator. Simulations have shown that DQ-MAC outperforms 802.15.4 MAC and BSN-MAC for all traffic loads in a generalized BSN scenario in terms of eliminating collisions while minimizing the control overhead and hence the overall energy consumption per bit.

Schedule-based approaches to node communications within WBANs have the overhead of synchronization, but have the advantage of being collision free, low duty cycle in operation and do not suffer from high levels of overhearing. Nodes can only transmit data during their assigned time slots, therefore, collisions are avoided. Nodes can turn their radio on for their assigned time slots ensuring that low overhearing and low duty cycle operations can be achieved. TDMA-based protocols can reduce latency by guaranteeing dedicated time slots for each node to send its data. HeartBeat MAC (H-MAC) [10] is a MAC protocol designed for WBANs that exploits heartbeat rhythm to perform time synchronization for TDMA. Although heartbeat is a key life-sign that can be monitored as part of a WBAN via an ECG sensor, nodes can use the heart rate waveform peaks as a mechanism for node synchronization within a WBAN. Nodes can achieve synchronization by using the heartbeat rhythm without having to turn on their radio. This means that the energy cost for time synchronization can be avoided thereby increasing the lifetime of the network dramatically. H-MAC has the obvious limitation of a single point of failure, *i.e.*, the reliance on the human heart for synchronization. Additionally, results may be affected by patients with a weak heart or those suffering from a cardiovascular condition where some sensor nodes may not be able to detect the synchronization data.

Sensor nodes may request varying channel resources under different scenarios. Steady state monitoring, for example EEG or blood glucose transmissions, could yield channel priority to heart rate and ECG sensor data which depending on the criticality of the patient, could be classified as high priority. Liu *et al.* [48] presented Context Aware MAC (CA-MAC) that is able to adopt different transmission strategies depending on the variation of patient activity, vital life signs, or environment status. The protocol is designed around *hybrid frame structure*, *channel aware adjustment of access mechanisms*, and *traffic aware adjustment of transmission priority*. The protocol incorporates a hybrid approach to channel access using a TDMA and contention-based model to reduce energy consumption and latency. The dynamic adjustment of channel access in CA-MAC can significantly increase the probability of reduced packet loss and ensure successful transmission. Based on traffic requests, sensor nodes with high priority can access the channel faster and transmit more data whereas more steady state low priority traffic is restricted. This approach means that priority nodes obtain a higher duty cycle and are allocated more slots for data transmission. Lower priority nodes can release available bandwidth or even cease transmission thereby preserving battery life. Simulation of CA-MAC showed a packet loss rate of 50% lower than comparable MAC protocols with a reasonable tradeoff between reliability and efficiency.

Al Ameen *et al.* [49] presented a WBAN specific TDMA based power efficient MAC using an on-demand wakeup radio mechanism. An additional receiver attached to the sensor node operates independently from the main node radio to reduce idle listening and reduce power consumption at the node level. The model incorporates periodic and emergency traffic scenarios. The proposed MAC is compared against B-MAC, X-MAC, WiseMAC and ZigBee (802.15.4) and is shown to offer improvements in terms of power efficiency and delay in single hop scenarios. Kwak and Ullah [50] also used a separate wakeup radio mechanism as part of their Traffic-adaptive MAC (TaMAC). WBAN traffic was categorized into *normal, emergency and on-demand* traffic types. The main radio dealt with normal traffic and the second was used for emergency/on-demand traffic. Results showed improvements over 802.15.4, WiseMAC and S-MAC in terms of power consumption and delay.

Hussain *et al.* [51] developed a TDMA based directional MAC with multi-beam directional antenna to facilitate simultaneous transmission in different directions in a single time slot at the same frequency. The protocol caters for the scenario where the WBAN differentiates between normal and urgent traffic by the use of two BAN coordinators. Urgent packets are directed to a secondary BAN coordinator when the node doesn't have its own guaranteed time slot (GTS). A summary of the WBAN MAC protocols is shown in Table 3.

### 5.4. Network Layer

In a traditional WSN context the network layer has to cater for numerous disparate nodes potentially spread over a wide geographical area. Network layer protocols make use of routing algorithms that use multi-hop to route packets to the destination node. In comparison to WSNs, WBAN nodes are mobile, likely to be clustered in specific areas and do not have the same extensive multi-hop requirement as WSNs. In comparison to WSNs, the bandwidth available to WBANs is limited, likely to be shared with other local ISM band devices and is subject to physical layer attributes such as interference, fading, and attenuation due to the specific characteristics of the human body. Existing WSN research at the network layer doesn't fully consider the range of heterogeneous nodes within a WBAN and are generally concerned with energy efficient routing via static nodes rather than the extensive body movements in a WBAN. The required routing algorithm complexity within WBANs is low in comparison to large-scale WSNs where nodes are potentially distributed across large-scale areas. However, the unique factors relating to communicating in and around the human body mean that network layer issues need to form an important part of WBAN protocol design.

The standard WBAN architecture of a number of heterogeneous nodes linked to a BCU or sink device often assumes single hop routing where all node communications are transmitted directly to the BCU. Zawowski *et al.* [30] looked at the potential of multi-hop in a UWB based WBAN with an energy comparison for a transmission from a node located on the back of a patient to a node on the chest. Results showed that a multi-hop strategy is recommended if the energy for pulse generation and reception is higher than a ten thousandth part of the energy for bit encoding and decoding. The criteria to use multi-hop depends on the ratio of the energy consumption needed for decoding/coding and receiving/generating a UWB pulse. In experiments to evaluate path loss models for human body parts (torso, back, arm, leg) Reusens *et al.* [31] and later Joseph *et al.* [52] attempted to identify whether single or multi-hop transmissions are preferable in a WBAN. Observations in both showed that for nodes furthest away from the sink, there is scope for additional energy saving as these nodes consume the most energy and will die first. However, in the multi-hop scenario, the nodes closest to the sink consumed more energy as these nodes need to forward the data received from nodes further away. Results showed that a smart combination of single and multi-hop routing could yield the optimum energy efficiency.

Implanted nodes as part of a WBAN can create a thermal effect due to the properties of RF signal communications in and around the human body. Specific Absorption Rate (SAR) is a measure of the rate at which radiation energy is absorbed by tissue per unit weight. Exposure to high levels of SAR could result in tissue damage. Tang *et al.* [53] proposed a Thermal Aware Routing Algorithm (TARA) that handled packet transmission in the presence of temperature hot spots by effectively routing round areas that exceeded temperature thresholds. Results showed it to be a safer routing solution whilst balancing transmission delay and less network congestion. Adaptive Least Temperature Routing (ALTR) [54] improves upon the performance of TARA by routing packets to the least highest temperature node. Least Total Route Temperature (LTRT) protocol [55] converts node temperatures into graph weights to generate minimum temperature routes. Since LTRT aims to send packets with the shortest hop counts, it prevents the entire network temperature from rising quickly. Results showed that packets required less hop counts in comparison with LTR and ALTR. Kamal *et al.* [56] proposed a lightweight rendezvous algorithm (LR) that divides nodes into small clusters and showed that LR generated lower levels of temperature than LTRT and ALTR.

The clustering of nodes, where energy consumption from communications with the sink is equally divided between each of the nodes in the cluster, has been shown to deliver performance improvements in WSNs and could have potential for WBANs. One of the first protocols to demonstrate the advantages of clustering was the Low Energy Adaptive Clustering Hierarchy (LEACH) protocol [57]. LEACH introduces data fusion into the routing protocol to reduce the amount of information that is transmitted to the sink to deliver significant improvements when compared to conventional routing protocols. Qin *et al.* [58] highlighted that LEACH and many of its successors assumed that all nodes in the network can communicate with the sink directly at all times and that this can be inconsistent with the practical application. Qin *et al.* proposed a Balanced Energy Consumption and Cluster based Routing Protocol (BECCRP) that improved upon LEACH where it sets gateways to relay the data from the cluster heads to share the energy consumption equally at each node, thereby extending the overall network lifetime. The protocol however, did not consider the nodes location and distance from other nodes. The summary of the performance of network protocols for WBAN is shown in Table 4.

### 5.5. Transport Layer

Transport layer protocols operating in a WBAN need to offer reliable packet delivery from the node to the BCU and in the case of specific sensor queries or emergency sensor data requests, reliable data delivery from BCU to node. Another key function of a transport protocol is the management of congestion in the network. Efficient control of network congestion can conserve battery life and increase the efficiency of packet delivery. Traditional transport layer protocols such as Transmission Control Protocol (TCP) and User Datagram Protocol (UDP) are too heavyweight and complex for WBAN applications resulting in latency and excessive energy wastage for low power wireless networks. TCPs 3-way handshake process with its end-to-end ACK approach would result in increased delay, increased buffer storage demands at the node level and ultimately poor QoS levels. UDP, although a connectionless protocol, has no guaranteed levels of reliability and will simply drop packets with no potential for recovery, this would obviously be a serious problem for life signs monitoring via a WBAN.

The design of energy efficient transport protocols needs to take into account the diversity of applications, traffic characteristics and resource constraints (namely energy and fairness) amongst a number of heterogeneous nodes. Protocols need to provide end-to-end reliability, QoS in an energy efficient way and be evaluated using metrics such as Packet Loss Ratio (PLR), latency, and fairness. Therefore, transport protocols should have components for congestion control and loss recovery since these two components have a direct impact on energy efficiency, reliability, and QoS [59].

Examples of transport layer protocols include COngestion Detection and Avoidance (CODA) [60], Pump Slowly Fetch Quickly (PSFQ) [61] and Event to Sink Reliable Transport (ESRT) [62]. CODA is a congestion control protocol that provides local congestion mechanisms to provide both buffer occupancy level and channel load condition to indicate network congestion. PSFQ provides reliable transport from sink to sensor (so called reverse path). PSFQ relies on the pump slowly operation to limit congestion but congestion will occur as the node numbers increase. A design solution involving a combination of CODA and PSFQ could potentially provide both congestion control and reliability in low power sensor networks. ESRT aims to provide reliability and congestion control with minimum energy usage. Reliability is defined by the number of data packets originated by an event that is successfully received at the sink using the ESRT algorithm. The ESRT algorithm tracks the event reporting frequency (*f*) of the received packets and matches it with the required reliability metric. Congestion is managed by decreasing reporting frequency without affecting reliability.

### 5.6. Application Layer

The application layer for a WBAN includes the application running on the node that may be specific to the sensor type as well as management, security, synchronization, and query type functions. Any application specific QoS constraints, e.g., latency, are handled at this level together with any data compression and signal processing. Although extensive research has been undertaken into protocols operating within the lower layers for WSNs/WBANs, there is very little existing research for protocols operating at the application layer.

At the application layer system administrators can interact with the nodes by using a Sensor Management Protocol (SMP) [63]. A SMP enables the lower layers to transparently interface with the application layer to undertake key management tasks such as management of rules relating to attribute naming and clustering, time synchronization and authentication. Sensor Query and Data Dissemination Protocol (SQDDP) [63] provides interfaces to issue attribute based queries. Sensor Query and Tasking Language (SQTL) [64] provides a mechanism to interface with events generated by a sensor node.

Compression of data at the application layer is a vital process that consolidates the data contained within each packet therefore, requiring fewer transmissions resulting in energy savings. Distributed Source Coding (DSC) [65] is a widely referenced compression technique used in WSNs. Nodes located close to each other may sense values that do not differ significantly. Each node would therefore, encode its data using fewer bits than it ordinarily would, making it impossible to retrieve the measured value with information from only one node. However, when all measurements reach the sink node, the DSC technique can combine the sensors encoded data to retrieve the values measured by all the sensors with minimal coding error. This process effectively moves the computational complexity from node to sink, thus fewer bits are transmitted and energy is saved. Other techniques such as Data Fusion and Data Aggregation [66] are further examples of methods used to reduce transmission data and save energy in low power networks.

Compressed Sensing (CS) is a relatively new signal processing technique that holds great promise for leveraging greater efficiency in the compression and recovery of signal data [67]. CS theory asserts that sufficient signal data can be recovered from far fewer samples or measurements than traditional methods where the sensed data adheres to a number of key criteria. CS relies on two principles: *sparsity*—which relates to the signal of interest and *incoherence*—which relates to the sensing modality [68]. Efficient sampling and sensing protocols can be designed based on CS techniques to capture useful data content embedded in a sparse signal and then condense the raw data into a reduced data set. What is most remarkable about CS is that it will allow a sensor node to very efficiently capture data in a sparse signal without needing to comprehend that signal, then by using numerical optimization techniques, CS can reconstruct the full length signal from the small amount of collected data. CS is a very efficient and effective signal acquisition method that in the context of a WBAN, could sample the data from a sensor node at a low rate and later reconstruction from what appears to be an incomplete set of measurements at the sink. The complexity of CS in comparison to standard Nyquist rate based signal processing techniques is moved to the sink side. This process alleviates complexity and therefore, battery draining processing from the sensing node. CS is an active research area and could hold great potential for WBANs, but careful analysis of the recovered signal must form a vital part of any research to ensure no QoS thresholds are breached and the accuracy of patient life signs data is not compromised.

## 6. The Case for Cross-Layer Design

### 6.1. Introduction

Cross-layer design is the concept of merging two or more layers within the protocol stack to improve the efficiency of the interaction between the protocols. The Open Systems Interconnection (OSI) model is a protocol stack abstraction that was developed to standardize communications by developing the concept of logical layers. The protocols at each layer hide the complexity of the layer below and provide services to the layer above. The protocols that fit into this type of model do not need to be aware of other protocols in the layers above or below. This offers many advantages in terms of scalability, compatibility, and flexibility of network design.

Resource efficiency can be gained by exploring a more unified scheme that merges common layer protocol functionalities into a cross-layer module for resource constrained nodes. However this type of structure, whilst possessing many advantages, cannot offer a consistent one size fitting solution for all network design problems. Protocol schemes that move away from the traditional layered model, whilst identifying significant advantages, must counter these improvements in network performance with a balanced view of the potential drawbacks of scalability or compatibility that may be introduced with the implementation of cross-layer solutions. The interaction between the layers can be varied but the justification to move away from the traditional model must be based on key benefits with a careful assessment of any downsides.

### 6.2. Justification for Cross-Layer Design

There are a number of reasons why the traditional layered approach to network design has been questioned and many of these have direct relevance to the specific needs of WBANs:

#### Network Heterogeneity

The standard layered protocol stack can adequately cater for the requirements of heterogeneous networks such as WBANs in terms of integration into a defined structure, but this may be at the expense of performance and energy efficiency. For example if a routing path is set up between two nodes through different types of network, the routing protocol can simply find an available path considering metrics as shortest distance or lowest cost. However, if nodes want to find a path with enough bandwidth, then the routing protocol needs to consider the interactions with the MAC layer. This cross-layer interaction isn't possible via the standard protocol stack.

#### QoS

Due to their application, WBANs have a specific requirement for high levels of QoS. High PDR, low levels of packet transmission delay, minimal collisions and retransmissions, optimal balancing of high energy efficiency and reliable transmission are all areas that are keys within the WBAN environment. The traditional layered structure may not be the optimal solution to guarantee these critical levels of service and may limit the potential improvements needed to guarantee energy efficiency and improvements to QoS.

#### Channel Conditions

The capacity of a link within a WBAN can vary not just due to the standard wireless link issues such as node interference [13], but issues due to the inherent behavior of RF signals in and around the human body such as signal absorption and attenuation effectively resulting in a variance in RF transmission power and path losses depending on the direction of propagation. Channel conditions can change due to factors such as antenna orientation, clothing type, physical stature of the patient, and postural position [69]. These changes in channel conditions can be catered for specifically within cross-layer schemes that incorporate link adaptation to take account of channel variances.

#### Performance

The closely coupled nature of the standard layered protocol stack means that interaction between protocols is standardized but potentially at the expense of performance. Data communication flows from one layer to another regardless of whether the protocol in any particular layer has any significant role to play in the overall transmission or receipt of data.

### 6.3. Methodology

Most cross-layer schemes involve the communications between different adjacent or non-adjacent layers of the protocol stack to improve the performance in some way over the traditional layered approach. These performance gains can be in the form of energy efficiency, reduced contention, better routing decisions or improved reliability. Research has shown that cross-layer designs can have a beneficial effect on the performance of wireless networks [70–72]. Taking a step back and looking at the protocol stack as a whole can help to focus on new solutions to wireless communication issues that a cross-layer approach can alleviate. Srivastava *et al.* [73] examined cross-layer interactions and summarized the various proposals in terms of methods that violate the traditional layered architecture. The main categories studied were: *Creation of new interfaces:* schemes that connect lower and higher layers either up or down and schemes that create an iterative flow between the two connected layers in both directions. *Merging of adjacent layers*: two adjacent layers merge their functionality to create a union of services. *Vertical calibration across layers*: setting of parameters across the layers either at design or runtime. *New abstraction that replaces the protocol stack*: disposing of the traditional protocol stack and replacing it with a new method that performs the same functions but in a non-layered form.

#### 6.3.1. Creation of New Interfaces

Performance gains can be achieved where a higher layer can use a parameter that is available in a lower layer to make better, more informed choices at the higher level. For example a routing algorithm at the network layer could select a route based on a channel condition parameter, e.g., CCA, at the physical layer or the MAC layer adapting the channel in terms of modulation or code rate, based on the channel quality at the physical layer. Lower layers can also be linked to higher layers in a scheme where the information flow is downward through the protocol stack. Here for example, the application layer could directly link with the MAC layer to communicate upon latency requirements as part of an overall QoS requirement, allowing the MAC layer to treat these packets as a priority [74].

#### 6.3.2. Merging of Adjacent Layers

Although there are numerous cross-layer schemes that couple, for example, the MAC and physical layers [75,76], these schemes still retain the traditional stack architecture. Efficiencies can be gained by creating a new super-layer that effectively merges the functions of both layers without the traditional overhead of two entirely separate layers. This union of the two layers services would not require new stack interfaces but rely on the existing interfaces to the rest of the protocol stack.

#### 6.3.3. Vertical Calibration across Layers

This type of cross-layer interaction involves the adjustment of parameters that span different layers in the protocol stack. The motivation here is that the potential performance gain at any particular layer is a function of all the parameters of the layers below it. Therefore, joint optimization undertaken in this way can yield increased performance benefits over optimization at the individual layer level. To enhance throughput, Adaptive Modulation and Coding (AMC) has been incorporated at the physical layer to match time varying channel conditions to transmission rates. Achieving high reliability at the physical layer however, requires protocols that can reduce transmission rates using either small size constellations or powerful low rate error control codes [77]. Another way to mitigate channel fading is to utilize ARQ at the MAC layer to improve system throughput and to limit the number of retransmissions. Lui *et al.* [77] considered AMC at the physical layer and ARQ at the MAC layer as part of a joint cross-layer design that was able to outperform AMC and ARQ working in isolation within their respective layers.

#### 6.3.4. Layering as Optimization Decomposition

This method of cross-layer design effectively dispenses with the traditional protocol stack and replaces it with a completely new architecture. Layering as optimization decomposition is a concept that effectively decouples the traditional protocol stack and provides a framework for cross-layer design [78]. In this method the traditional protocol stack is dispensed with and replaced by a single integrated solution. The research by Chiang *et al.* [78] positions this theory as carrying out an asynchronous distributed computation over the network to implicitly solve a global optimization problem modeling the network. The theory exposes the interconnections between layers as different ways to modularize and distribute a centralized computation. Separate vertical decompositions (functions) of an optimization problem in the form of a generalized network utility maximization (NUM) are mapped to a layer or the interfaces between specific layers. The NUM can take the form of vertical or horizontal decomposition. *Vertical decomposition*: entails the decoupling of the protocol stack where network protocol functions such as energy detection, congestion management, routing, error control etc are coordinated as part of modules that perform the same functions as the separate layers. *Horizontal decomposition*: can be carried out to further optimize and control the functional layer interaction. The net effect of this is to decompose the optimization problem into sub-problems each associated or mapped to a specific protocol layer with defined functions managing the interfaces between the layers. Layering as Optimization Decomposition presents a “clean sheet” approach to the design of a protocol stack. Chiang *et al.* positions the theory as being outside of and separate to the existing research on cross-layer design. However, Layering as Optimization Decomposition does not supersede the need for cross-layer design. The decomposition process involves the development of modules that retain coupling between layers and could be seen as justifying the requirement for cross-layer design [79]. The need for cross-layer design solutions is reinforced in cases of architecture mismatch between an existing protocol stack and an optimal decomposition method. Layering as Optimization Decomposition provides a top down approach to designing network architecture from first principles. The resulting conceptual simplicity stands in contrast to the ever increasing complexity of communication networks [80]. Although the case for Layering as Optimization Decomposition is still to be fully made in terms of its practical application, it can provide benefits for the modeling of a decomposition scenario in providing a direct comparison to an existing protocol stack identifying which layers to integrate.

## 7. Cross-Layer Design Schemes

### 7.1. Introduction

Cross-layer protocols design specific to WBANs is a relatively immature research area but has the potential to deliver greater efficiencies than single layer adaptation schemes. Cross-layer protocol research in the comparable WSN area is more established with many schemes able to demonstrate significant energy efficiency improvements via the optimization of two or more layers. Generally, cross-layer schemes can be divided into *loosely coupled* and *tightly coupled designs*. Loosely coupled protocol designs focus on adapting the parameters available at a lower layer to optimize the performance at a higher layer. For example channel conditions at the physical layer could be utilized at the network layer as part of an intelligent routing algorithm. In the loosely coupled approach the individual layers within the protocol stack remain, but the parameters normally accessible only to the next highest layer could be made available to higher layers to provide performance gains or better/more informed decision making at the upper layers. In the tightly coupled approach to cross-layer design, the different layers are optimized together to form one complete solution to an optimization problem. Performance gains for tightly coupled designs should be greater than loosely coupled as they do not incur the same stack communication overhead, however this may be at the expense of protocol transparency and maintenance. This section highlights the benefits of cross-layer interaction across specific layers and details some of the relevant cross-layer schemes and the benefits they deliver to low power wireless communications.

### 7.2. MAC and Physical Layer Schemes

As the physical and MAC layers are close to each other in terms of their protocol stack roles, many cross-layer schemes have adapted both layers as part of a jointly optimized scheme to deliver improvements to traditional protocols. In practice, protocols generally incorporate a closely coupled interaction between the lower parts of the MAC layer and the physical layer. Many physical layer techniques for transmission, modulation and spread methods can enhance performance at the physical layer by improvements in throughput, delay and collision management, but it's only at the MAC layer level that these performance gains can be best adapted and optimized. Therefore, a protocol that incorporates the cross-layer design at the MAC/physical level has the potential to deliver enhanced performance over the traditional separate layer structure.

A variety of factors can affect performance in a low power wireless network, such as channel properties, traffic load, energy management policy, *etc*. The time varying nature of the wireless channel can have a significant influence on node energy consumption where poor link quality can result in increased energy usage. The Channel Adaptive Energy Management (CAEM) protocol [81] utilizes the time varying property of the wireless link by exploiting the cross-layer interaction between physical and MAC layers to deliver performance gains of up to 30% when compared to traditional protocols. CAEM allows a node to dynamically adjust the data throughput by changing the amount of error protection incorporated by the node according to the current quality of the link, estimated bandwidth and traffic load. The protocol buffers the packet temporarily until the channel recovers to the required quality. The obvious overhead is the inherent latency and potential buffer overflow due to the temporary storing of packets. To avoid this the protocol incorporates a scheduling and queuing algorithm to ensure that every sensor can equally access the wireless channel under such a fluctuating environment, thus achieving a balance between energy efficiency and fairness.

Many link adaptation schemes use the Signal to Noise Ratio (SNR) as the only input from the physical layer. For frequency selective channels, however, the SNR alone does not adequately describe the channel quality. Cross-layer performance can be significantly improved if the link adaptation scheme is based on a more detailed description of the channel condition. An extended exchange of information between the MAC and physical layers is required to fully exploit the status of the current channel conditions [82]. Utilizing detailed knowledge of the actual channel condition that is already available at the physical layer as prediction of the packet error probability for the currently observed channel can be obtained and exploited to improve performance. At the physical layer the parameters *transmit power, modulation, and coding rate* have a direct impact on multiple access of nodes in wireless channels by affecting node interference. Local adaptation of these parameters to achieve a target Bit Error Rate (BER) restrains both routing and MAC decisions.

Kozat *et al.* [71] considered end-to-end QoS guarantees at the physical and MAC layers and proposed a cross-layer framework that highlighted the gains in closely integrating these two layers. QoS can be interpreted differently at each of the protocol layers. At the physical layer, QoS can be defined as an acceptable BER or Signal to Interference to Noise Ratio (SINR). At the MAC layer and higher layers, it is normally expressed in terms of minimum rate or maximum delay guarantees. A QoS guarantee that is explicitly based on both minimum rate requirements and maximum tolerable BER is a good compromise, but must be satisfied at minimal energy expenditure. Wang *et al.* [83] developed a cross-layer design for healthcare monitoring that combined two modules: AMC in the physical layer and two sleep modes in the MAC layer. Normally AMC is used to change the transmission rate at the physical layer, improve BER, or vary bandwidth. Time slotted and unslotted sleep modes were used in the MAC layer. Energy consumption was expressed by the product of power level and time slot length. Simulation results showed that the cross-layer scheme achieved minimal energy consumption, thereby extending node lifetimes, while the spectral efficiency in the whole SNR range was maximized.

### 7.3. Network and MAC Layer Schemes

The network layer is responsible for selecting the optimal routing path for data packets. Different routing decisions alter the set of links to be scheduled and thereby influence the performance of the MAC layer, e.g., if the routing protocol chooses flow paths that are closer to each other, the subsequently higher interference and potential contention in the network make it harder for the MAC to resolve the transmission conflicts. When QoS requirements are ignored and link costs that accurately quantify the energy consumption can be assigned, the network layer becomes the sole determinant of energy consumption [71]. These link costs, however, depend on the transmit power which is a function of decisions made at the MAC and physical layers.

In many areas of adaptive MAC protocol design: S-MAC [33], T-MAC [35], and DMAC [36], the research is focused on the adaptation of the MAC layer and ignores the inter-working between different layers thereby attempting to avoid collisions among neighbor nodes without considering any routing information from the network layer or traffic patterns from the application. The best routing path can be identified by extracting key information from the lower layers, such as traffic volume, link quality, and collision data, to enhance performance at the network layer [84–87]. It could be argued that integrating routing with MAC as part of a cross-layer jointly optimized solution makes sense as the two functions are very closely coupled and a case can be made for integrating the layers either as one complete module or two closely coupled modules in the same layer. If routing is not taken into account by the MAC layer, optimal performance can only be achieved locally [80].

Cui *et al.* [88] proposed that energy efficiency must be supported across all layers of the protocol stack through cross-layer design. The proposed scheme incorporated a variable length TDMA protocol where the slot length is optimally assigned depending on the network layer routing requirement while minimizing the energy consumption across the network. CoLaNet [89] considered characteristics of the application to make better routing path choices at the network layer and demonstrated energy savings over S-MAC. Safwati *et al.* [75] proposed schemes to utilize cross-layer interactions between the MAC and network layers where the MAC sublayer provides the network layer with information pertaining to successfully receiving a CTS or an ACK frame or failure to receive one. This allows the network layer to choose the route that minimizes the probability of error to achieve the most energy effective route.

Most cross-layer designs look at the integration of adjacent or non-adjacent layers to adapt the protocol stack to deliver efficiencies over the traditional stack architecture. Schemes that extend the optimizing techniques to layers of intermediate nodes can provide additional energy saving benefits [70]. The network layer of a source node could be made aware of the channel conditions in the lower layers of the nodes in the neighborhood as part of a multi-hop scenario. Nodes could detect congestion and interference at the MAC layer and immediately react to it at the network layer. Routing decisions could be better addressed as more knowledge about the MAC and physical layers of adjacent nodes is available. Ruzzelli *et al.* [76] proposed Timezone Coordinated Sleep Scheduling (TICOSS) protocol as a cross-layer energy efficient multi-hop protocol based on 802.15.4. The network was divided up into time zones where each one takes turn in the transmission where nodes in the farthest time zone start the transmission. In the next slot the farthest but one sends its data and so on until the sink is reached. The protocol mitigates the hidden node problem, provides configurable shortest path routing to the PAN coordinator and almost doubles node lifetime for high traffic scenarios compared to standard WSN protocols.

Any protocol operating at the network layer needs to resolve the inherent conflict between energy efficiency and throughput. Sleep Collect and Send Protocol (SCSP) [90] dynamically calculates the node sleep and data receive (collect) periods depending on the amount of incoming traffic. The network layer uses a modified version of ZigBee routing that is more tolerant to node failures. The MAC layer provides the list of neighbor nodes to the network layer, which in turn provides multiple forwarding choices to it. Therefore, the MAC protocol has the option to change to the next hop router during transmission if the message is not successfully transmitted after *n* number tries. SCSP saves energy consumption by switching between active and sleep periods by dynamically adapting them depending on the amount of received traffic. The protocol uses a simple but efficient routing protocol that does not need route maintenance or discovery and works jointly with the MAC layer to enhance its fault tolerance properties. Simulations show that SCSP extends the network lifetime and connectivity in comparison with 802.15.4.

As the channel quality can vary due to the changing characteristics inherent in the physical layer of a wireless network, resource at the MAC layer is therefore, variable [80]. These variations can result in fluctuations of link capacity and transmission since transmission rate is related to many factors, not just link quality. Joint optimization for rate control coupled with resource allocation and routing in the context of multichannel operation is a complex challenge for network/MAC designers.

### 7.4. Transport and MAC, Physical Layer Schemes

Due to energy constraints and protocol simplicity, some low power standards, e.g., ZigBee that overlays 802.15.4, offers only best effort end-to-end delivery of individual packets. This means only limited transport layer functions (packet fragmentation and re-assembly) at the application layer as there is no fragmentation support at the network layer. Other standards that are designed to underpin the higher protocol layers require transport layer functions that overlay a low power MAC/physical to implement a full protocol stack.

Typically for traditional wireless networks, the standard protocols operating at the transport layer are TCP—*providing connection orientated services to the network*, and UDP—*providing connectionless services*. Overlaying a transport layer protocol such as standard TCP or UDP over a WBAN/WSN structure can be problematic in that each has their own overheads that impact on the energy efficiency of low power networks. For example, transmitting IPv6 packets over 802.15.4 cannot take place without the inclusion of an additional transport layer between IPv6 and the 802.15.4 MAC layer that basically compresses the frame to meet the physical payload size required by the protocol. Standard UDP has no mechanism for reliable communication, therefore cannot be considered in the WBAN context, as there is no method to support QoS. Additionally, as UDP cannot control traffic rate at the transport layer. The only way the traffic can be managed is via connection admission control or end-to-end rate control. These schemes would need to be cross optimized with the physical layer to cater for the variable link capacity.

Standard TCP can manage congestion avoidance, but as it was originally designed for wired networks, it suffers from performance issues related to interpretation of BER, limited bandwidth, and assumptions that packet loss is mainly caused by congestion. Performance gains can be made by optimizing a congestion control algorithm utilizing physical layer parameters as part of a cross layer scheme.

As the wireless link is prone to varying channel quality, fading, and interference, transport layer protocols can deliver better performance when optimized to consider the varying link capacity [91]. Cross-layer design solutions operating at the transport and physical layers could, for example, report the channel condition from the physical layer as a new parameter to the transport layer that would enable a protocol to distinguish real congestion from packet loss to make better performance related decisions. There is a direct relationship between the physical/MAC layer and transport layer where adapted cross-layer protocols can deliver higher throughput and minimal end-to-end packet delay. Spectrum sensing mechanisms operating at the lower layers can mitigate the issues relating to neighbor node interference, allowing protocols to sense the spectrum and access the channel in an opportunistic way allowing the transport layer to make more informed decisions concerning congestion and collisions in delivering reliable communications [ 92].

### 7.5. Application and MAC, Physical Layer Schemes

Although there are numerous examples of cross-layer schemes involving the lower layers, generally, cross-layer solutions incorporating the application layer do not figure highly in the available literature. Many existing protocols operating at the application layer were designed for wired networks and do not perform optimally in the low power wireless context. Schemes that can perform application level adaptation based on information from lower layers could provide performance benefits in low power wireless networks. The application layer can communicate the applications' QoS needs, *i.e.*, data reliability, delay tolerance and response time to other layers [93]. Additionally, information on channel conditions from the MAC/physical layers could be used by the application layer as part of a cross-layer solution to improve performance. Rahman *et al.* [94] developed an adaptive cross-layer mechanism to control congestion for real- and non-real time data flow to support QoS guarantees at the application layer. Priority was given to real time data in terms of delay and available link capacity. The scheme linked the QoS requirements at the application layer and the packet waiting time, collision resolution and packet transmission time metrics at the MAC layer. Correlation-based Collaborative MAC (CC-MAC) protocol [95] is a cross-layer solution incorporating the application and MAC layers. CC-MAC exploits the spatial correlation between nodes to reduce energy consumption without compromising the estimated reliability achieved at the sink. Experiments showed that CC-MAC delivered improved performance over S-MAC and T-MAC in terms of energy efficiency, packet drop rate, and latency.

In their work on a new energy saving MAC model, Otal *et al.* [47] developed a cross-layer solution incorporating a fuzzy rule scheduling algorithm and radio activation policy. The researchers present Distributed Queuing Body Area Network (DQBAN) MAC as an enhancement to 802.15.4 for a BSN that extends the research carried out by Lin and Campbell [96]. The research incorporates a fuzzy rule scheduler that optimizes the MAC layer to improve overall performance in terms of QoS and energy consumption. The protocol considered the node cross-layer constraints such as physical layer signal quality (SNR), system waiting time, and residual battery life to allocate slots within a superframe structure. Simulations covering homogeneous and heterogeneous scenarios demonstrated that DQBAN MAC can achieve higher reliabilities when compared to 802.15.4 whilst delivering specific latency demands and battery limitations.

As part of research that focused on the benefits of cross-layer optimization between application and MAC layers, Chatterjea and Havinga [97] illustrated how two examples of an adaptive MAC could benefit in exchanging information with the application layer. To solve the problems of rapidly draining power sources and congestion in the network, the research exploited the high degree of spatial correlation that exists between the readings of adjacent nodes in a densely deployed network by utilizing a distributed and self organizing scheduling algorithm. The scheduling algorithm prevented two adjacent nodes acting as correlating nodes simultaneously and increased the robustness and accuracy of the data by giving every node a chance to act as a correlating node. Although simulations showed energy savings of up to 42% when using the cross-layer information, the researchers recognized that disturbances in the lower layers could have a detrimental effect on the upper layers. In particular, changes in link quality may lead to a reduction in the efficiency of the algorithm owing to the continuous rearranging of the scheduling scheme. This highlights the need for the upper layers to be adequately insulated from the instability of the lower layers.

Gaudadri and Baheti [98] proposed an application and MAC cross-layer solution for packet loss mitigation for WBANs using CS based on ECG signals. The work addressed the issues of end-to-end packet losses due to excessive path loss, interference, handoffs, congestion, and system load. It was observed that when the signal contains redundancies, the packet losses can be treated as lossy compression performed by the channel. Lost packets are identified at the application layer via a sequence number field in the packet header of the lower layers. The research observed that reconstruction accuracy degrades gracefully as packet loss rate increases. ECG signals can be recovered with high fidelity even in high packet loss conditions with 99% heartbeat detection accuracy at packet loss rates of 20% with a constant latency of less than 2.5 s.

### 7.6. Tightly Coupled—Multi-Layer Cross-Layer Schemes

In a network with multiple nodes all contending for resources, if no arbitration is enforced, QoS constraints can be breached. The traditional solution to this problem is to use the transport layer to perform congestion control, find the best path via an effective routing algorithm at the network layer, and use of the MAC protocol to access the channel to give the best single hop performance [79]. However, this type of mechanism can never deliver optimal performance as the algorithms in the various layers are not optimized together. Joint optimization between the full protocol stack is the only way to deliver full network wide scheduling and congestion control.

Akyildiz *et al.* [91] presented a Cross-layer Module (XLM) that replaced the entire layered architecture by a single protocol where the objective is reliable communication with minimal energy consumption, adaptive communication, and local congestion avoidance. Lin and Shroff [99] developed a similar scheme studying the performance bounds of cross-layer congestion control. XLM is governed by the Initiative Determination concept where the cross-layer protocol performs receiver-based contention, local congestion control, and distributed duty cycle operation to deliver efficient and reliable communications. Each node has the freedom to decide on participating in communication and initiate transmission by sending an RTS packet to indicate that it has a packet to send. Upon receipt the node will decide whether to participate in the communication.

Results show that XLM outperforms the traditional layered protocol stack in terms of performance and implementation complexity. One of the major advantages of cross-layer design is implementation efficiency. In a traditional layered architecture, each layer has clear boundaries and packet overhead leading to computation delays due to the sequential handling of a packet through the protocol stack.

Cross-layer Protocol (XLP) [100] extends XLM and merges the functionalities of traditional MAC, routing, and congestion control into a unified cross-layer module by considering physical layer and channel effects, avoiding the need for end-to-end congestion control. A summary of the protocols discussed above is shown in Table 5.

### 7.7. Pragmatic Approach to Cross-Layer Design

Wired networks are effectively a set of well defined reliable communication links over a hard wired transmission medium. Wireless communications, however, are broadcast in nature, subject to interference and signal attenuation and are inherently unreliable, especially in the context of on body communications. The current layered protocol stack was developed with wired communication in mind and has been adapted for use in the wireless context as a best effort use of existing architecture [73]. The new modalities inherent in the wireless medium are not optimally catered for in the current layered protocol stack and inevitably require at least some level of cross-layer design to develop the best solution for wireless communications. Cross-layer designs usually improve performance but can do so at the expense of higher complexity in communication and computation, making complexity reduction an important issue. Kawadia and Kumar [101] argued that layered architecture can be optimal in most cases. Cross-layer design can potentially work at cross purposes and if designers are not careful, a negative effect on system performance is possible [79]. Issues that cross-layer design can introduce are:

#### Additional system complexity & lack of flexibility

Enhanced performance of cross-layer schemes is often demonstrated via simulations or prototypes, however actual implementation of these schemes highlights issues of introduced complexities and constraints due to modifying a number of protocols in different layers.

#### Protocol incompatibility

As the traditional protocol stack is violated with cross-layer design, the inherent compatibility and interoperability of protocols that operate in the protocol stack is breached. The integration effort required to adapting a cross-layer protocol to effectively connect to a standard protocol cannot be underestimated and may negate the proposed benefits of any cross-layer design effort.

#### Network protocol evolution constraints

The in-built independence of protocols operating within the traditional layered protocol stack means that they can be maintained and changed without disrupting other protocols in the stack. This is more problematic with cross-layer design where any change to a cross-layer protocol would need to be potentially coordinated across a number of different layers.

The trade-off between performance and architecture needs to be considered in the overall context of any network design. Cross-layer designs need to view the totality of design including interactions with other layers and consider the long-term architectural benefit of the proposed cross-layer approach. The law of unintended consequences is key here, cross-layer solutions that show performance gains in the minutiae but result in complexities at other levels of the protocol stack do little to further the advancement of network design [101]. In order to further advance the evolution of wireless communications, the traditional protocol stack architecture needs to be challenged, but only within a framework that sustains and balances improvements against any engineered complexities. The guidelines set out in [101] serve as a set of rules for cross-layer design, namely: (a) map out the interactions between the parameters as part of a dependency graph for the entire protocol stack, (b) consider timescale separation and stability to avoid conflict where two parameters are contending for the same resource at the same time, (c) ensure any implemented cross-layer design is structured, stable, maintainable and able to demonstrate significant benefits that justify the move away from the traditional architecture.

## 8. Open Research Areas

Although WBAN specific research is an active area, only a limited number of commercial solutions have been made available outside the research community. The proliferation of WBAN technology in particular protocols specific to the monitoring of vital life signs is dependent on breakthroughs and advances in a number of key areas:

### Efficient Low Power Protocols

Protocols that deliver extremely high levels of energy efficiency are required for successful WBAN implementations. The focus on this main challenge is likely to remain a key area of ongoing research due to the limitations of energy supply and longevity requirements especially for embedded nodes. Context aware protocols that differentiate between steady state and emergency traffic provide an interesting insight but further research is needed to move this area forward form the modeling/simulation arena to real life practical applications [48,50].

### Extended Battery Lifetime

The potential of micro fuel cells and nanowire battery technology could result in significant breakthroughs for WBAN architectures. The resulting increase in density could significantly extend battery lifetimes over existing lithium based power supplies. The reduced size of these new battery technologies will result in a smaller sensor node footprint as the battery is currently a major contributor to node size.

### Security

It is vital that the key personal data that is communicated via WBANs is protected to ensure its integrity is maintained. However, complex communication protocols are expensive in terms of computational effort and, therefore, energy usage. Future research in this area needs to identify adequate levels of information security whilst not compromising on energy efficiency.

### Nanotechnology based Sensors

Recent innovations in nanotechnology show that the development of tiny fully integrated sensor devices that include densely packed batteries, bio-sensors and nano-scale RF transmitters is now a reality. The mechanisms for communicating with these devices may require new approaches requiring new protocols to facilitate communications at the nano-scale.

### Energy Harvesting

The limitations of battery life are a key consideration in the design of efficient WBAN protocols. Extending battery lifetime via the harvesting of available energy resources can be a valuable method in extending the operation of WBAN sensors. The human body emits heat and also vibration during movement. These sources of energy could be scavenged as part of an energy efficient protocol implementation to extend network life.

### User Experience and operational Issues

Much of the existing research has focused on the technical elements of sensing and efficiently communicating data within a WBAN. However, for systems to gain acceptance in the user community, designers will need to cater for the requirements of a wide range of real life scenarios that do not currently feature heavily in the available research. This will include the ability of systems to operate efficiently within extreme environments, in areas with high levels of interference, and that are able to adapt and perform in alignment with peoples lifestyles.

### Integration of WBAN architectures and the Internet

The integration of WBAN specific low power protocols and IPv6 is key to the seamless working of systems using these technologies. Future research will need to focus on tighter integration and interoperability between these two areas to ensure efficient communications from low power network to the Internet.

## 9. Conclusions

In this study we have reviewed the existing research in the area of WBANs. We have provided an introduction to WBAN and an overview of WBAN standards and protocols operating at the different layers of the protocol stack. The study includes the relevant research in WBAN related cross-layer design schemes and concludes with a discussion of open research areas.

Research has shown that effective solutions can be designed for low power monitoring of patients using the traditional layered protocol stack. However, adhering to the constraints of the traditional protocol stack means that any designed solution cannot be optimal. The violation of the protocol stack can deliver benefits that cannot be attained by adhering to the traditional structure. The work presented in the previous sections has highlighted the performance gains that can be achieved with energy efficient cross-layer design. The health warning here is that these gains can come with drawbacks that, in practice, could cancel out any protocol design efficiencies. Obviously, the points made in [101] are key and the issues raised therein are important ones, but when performance gains can be made without introducing high levels of complexity, then benefits can be realized. The case for cross-layer design for WBANs is a strong one, especially within the human body medium where specific factors such as signal attenuation, signal losses, and body mobility need a clean sheet design perspective, free of any traditional layered constraints that inhibit performance gains from the onset. Various adapted and cross-layer schemes have been presented from existing research. It is recognized that WBAN-specific protocol design is an immature subject and that the case for energy efficient cross-layer design in the WBAN context currently relies upon the benefits highlighted in the existing research for WSNs. However, although many parallels exist between the two types of sensor network, further research into the benefits of WBAN-specific cross-layer design is needed whilst ensuring these benefits are balanced against any potential drawbacks.

## Figures and Tables

**Table 1.** Comparison between CSMA/CA and TDMA.

**Performance Metric**	**CSMA/CA**	**TDMA**
Power consumption	High	Low
Efficiency at light traffic	Good	Poor
Efficiency at high traffic	Poor	Good
Bandwidth utilization	Low	Maximum
Scalability	Good	Poor
Effect of packet failure	Low	Latency
Synchronization	Not applicable	Required

**Table 2.** Low power MAC protocol.

**Protocol**	**Protocol Summary**
S-MAC	Saves energy by switching between active and sleep states. Maintains a common sleep schedule between neighbor nodes in low duty cycle operations. Duty cycle needs synchronizing to a specific load which can affect performance.
T-MAC	Uses adaptive duty cycle to dynamically end active part of the cycle to reduce energy wasted on idle listening. Can suffer from idle listening, reduced throughput, and increased latency.
DMAC	Utilizes data gathering trees to solve interruption problem. Adapts duty cycle depending on load.
B-MAC	Incorporates preamble sampling to reduce wakeup period. Drawback is that all nodes need to listen to the long preamble.
WiseMAC	Uses preamble sampling to mitigate overhearing and reduce power consumption. WiseMAC suffers from a long preamble and has no mechanism to adapt to changing traffic patterns. For the same delay, preamble sampling lowered power consumption by 57% when compared to 802.15.4.
X-MAC	Uses reduced length preamble, early node acknowledgement resulting in increased energy savings. Offers flexible adaptation to both bursty and periodic data sources.
NCCARQ-WSN	Network coding based. Uses less control packets than traditional cooperative ARQ protocols. Up to 50% more energy efficient without compromising QoS.
NC-PAN	Uses a hybrid cooperative network coded ARQ technique. Performance gains of up to 35% compared to B-PAN and C-PAN.

**Table 3.** WBAN MAC protocols.

**Protocol**	**Protocol Summary**
MedMac	Allows nodes with ultra low data rates to save power by sleeping through beacons normally received to synchronize with the network. Achieved energy savings of up to 87% over 802.15.6 for the selected scenarios.
BodyMAC	Gives flexible bandwidth allocation to improve energy efficiency by reducing packet collisions, lowering transmission times, idle listening and control packet overhead. Efficient sleep mode used to reduce idle listening duration for low duty cycles. Demonstrates superior performance compared to 802.15.4.
BSN-MAC	Designed to exploit feedback information from nodes to deliver increased energy efficiency. Control algorithm enables the BSN coordinator to adjust parameters in the 802.15.4 superframe structure to avoid idle listening and achieve both energy efficiency and low latency on energy critical nodes.
DQ-MAC	Grants immediate access for light traffic loads (behaving as a random access mechanism) and moves to a reservation system for high traffic loads, eliminating collisions for all data transmissions. Delivers energy saving improvements over BSN-MAC and 802.15.4.
H-MAC	Exploits heartbeat rhythm to perform time synchronization for TDMA. nodes use heart rate waveform peaks for node synchronization. Nodes can achieve synchronization without having to turn on their radio. Energy cost for time synchronization can be avoided, thereby increasing the lifetime of the network. Limitation of single point of failure.
CA-MAC	Adopts different transmission strategies, depending on variation of patient activity, vital life signs or environment status. Protocol incorporates a hybrid mechanism for channel access using TDMA and contention-based model to reduce energy consumption and latency. Demonstrated packet loss rate of 50% lower than comparable MACs with reasonable tradeoff between reliability and efficiency.
Power Efficient MAC	On-demand wakeup radio mechanism. Additional receiver attached to the sensor node operates independently from main node radio to reduce idle listening and reduce power consumption. Model incorporates periodic and emergency traffic scenarios. Offers improvements in terms of power efficiency and delay in single hop scenarios compared to B-MAC, X-MAC, WiseMAC and ZigBee (802.15.4).
TaMAC	Adapted to cater for *normal, emergency and on-demand* traffic types. Main radio deals with normal traffic and second is used for emergency/on-demand traffic. Results showed performance improvements over 802.15.4, WiseMAC and S-MAC.
TDMA directional MAC	Differentiates between normal and urgent traffic using two BAN coordinators.Urgent packets are directed to a secondary BAN coordinator when the node doesn't have its own guaranteed time slot.

**Table 4.** Low power Network protocols.

**Protocol**	**Protocol Summary**
TARA	Handles packet transmission in presence of temperature hot spots by routing round areas that exceed temperature thresholds. Results demonstrate a safer routing solution whilst balancing transmission delay with less network congestion
ALTR	Improves on the performance of TARA by routing packets to the least highest temperature node.
LTRT	Converts node temperatures into graph weights to generate minimum temperature routes. Sends packets with the shortest hop counts and prevents the entire network temperature from rising quickly. Results showed packets required less hop counts in comparison with LTR and ALTR.
LR	Divides nodes into small clusters and generated lower levels of temperature than LTRT and ALTR
LEACH	Introduces data fusion into the routing protocol to reduce the amount of information transmitted to the sink. Deliver significant improvements when compared to conventional routing protocols.
BECCRP	Improved upon LEACH where it sets gateways to relay the data from the cluster heads to share energy at each node to extend the overall network lifetime. Doesn't consider node location and distance from other nodes.

**Table 5.** Low power cross-layer protocols.

**Protocol**	**Protocol Summary**
CAEM	Allows a node to dynamically adjust data throughput by changing levels of error protection at the node according to quality of the link, estimated bandwidth, and traffic load. Protocol buffers the packet until the channel recovers to the required quality. Performance gains of up to 30% compared to traditional protocols.
CoLaNet	Incorporates the characteristics of the application to make better routing path choices at the network layer and demonstrated energy savings over S-MAC.
TICOSS	Based on 802.15.4. Network is divided into time zones where each one takes turn in transmitting. Mitigates the hidden node problem, provides configurable shortest path routing to the BCU and almost doubles node lifetime for high traffic scenarios compared to other standard protocols.
SCSP	Dynamically calculates node sleep and data receive periods depending on traffic levels. MAC layer provides the list of neighbor nodes to the network layer, which in turn provides multiple forwarding choices to it. Switches between active and sleep periods by dynamically adapting modes depending on traffic levels. Uses a simple routing protocol that doesn't need route maintenance or discovery. Extends the network lifetime and connectivity in comparison with 802.15.4.
QoS Adaptive Cross-layer Congestion Contol	Incorporates an adaptive cross-layer mechanism to control congestion for real and non-real time data flow to support QoS guarantees at the application layer. Priority given to real time data for delay and available link capacity. Scheme links the QoS requirements at the application layer and packet waiting time, collision resolution, and packet transmission time metrics at the MAC layer.
CC-MAC	Cross-layer solution incorporating the application and MAC layers. Exploits the spatial correlation between nodes to reduce energy consumption without compromising reliability at the sink. Delivers improved performance over S-MAC and T-MAC in terms of energy efficiency, packet drop rate, and latency.
DQBAN	Incorporates a fuzzy rule scheduler that optimizes the MAC layer to improve overall performance for QoS and energy consumption. Considers node cross-layer constraints such as SNR, waiting time, and battery life to allocate superframe slots. Protocol achieves higher reliabilities compared to 802.15.4 whilst delivering specific latency demands and battery limitations.
XLM	Replaces the entire layered architecture by a single protocol where the objective is reliable communication with minimal energy consumption, adaptive communication, and local congestion avoidance. Each node has the freedom to decide on participating in communication. XLM outperforms the traditional layered protocol stack in terms of performance and implementation complexity.
XLP	Extends XLM and merges the functionalities of traditional MAC, routing and congestion control into a unified cross-layer module by considering physical layer and channel effects avoiding the need for end-to-end congestion control.
